# Optimizing prostate cancer accumulating model: combined PI-RADS v2 with prostate specific antigen and its derivative data

**DOI:** 10.1186/s40644-019-0208-6

**Published:** 2019-05-23

**Authors:** Yuan-Fei Lu, Qian Zhang, Wei-Gen Yao, Hai-Yan Chen, Jie-Yu Chen, Cong-Cong Xu, Ri-Sheng Yu

**Affiliations:** 1 Department of Radiology, Second Affiliated Hospital, School of Medicine, Zhejiang University, 88 Jie-Fang Road, Zhejiang, 310009 Hangzhou China; 2 Department of Radiology, Yangming Affiliated Hospital, School of Medicine, Ningbo University, Yuyao, 315400 Zhejiang China; 3 Department of Urology, Second Affiliated Hospital, School of Medicine, Zhejiang University, Hangzhou, 310000 Zhejiang China

**Keywords:** Prostate cancer, MRI, Prostate-specific antigen, Model

## Abstract

**Background:**

To establish a new accumulating model to enhance the accuracy of prostate cancer (PCa) diagnosis by incorporating prostate-specific antigen (PSA) and its derivative data into the Prostate Imaging-Reporting and Data System version 2 (PI-RADS v2).

**Methods:**

A total of 357 patients who underwent prostate biopsy between January 2014 and December 2017 were included in this study. All patients had 3.0 T multiparametric magnetic resonance imaging (MRI) and complete laboratory examinations. PI-RADS v2 was used to assess the imaging. PSA, PSA density (PSAD), the free/total PSA ratio (f/t PSA) and the Gleason score (GS) were classified into four-tiered levels, and optimal weights were pursued on these managed levels to build a PCa accumulating model. A receiver operating characteristic curve was generated.

**Results:**

In all, 174 patients (48.7%) had benign prostatic hyperplasia, and 183 (51.3%) had PCa, among whom 149 (81.4%, 149/183) had clinically significant PCa. The established model 6 (PI-RADS v2 + level of PSAD + level of f/t PSA+ level of PSA) had a sensitivity and specificity of 81.4 and 84.5%, respectively, at the cut-off point of 11 in PCa diagnosis. Correspondingly, at the 12 cut-off point, the sensitivity and specificity were 87.7 and 83.0%, respectively, in diagnosing clinically significant PCa. The score of the new accumulating system was significantly different among the defined GS groups (*p* < 0.001). The mean values and 95% confidence intervals for GS 1–4 groups were 10.20 (9.63–10.40), 12.03 (11.19–12.87), 14.12 (13.60–14.64) and 15.44 (15.09–15.79).

**Conclusions:**

A new PCa accumulating model may be useful in improving the accuracy of the primary diagnosis of PCa and helpful in the clinical decision to perform a biopsy when MRI results are negative.

## Background

Prostate cancer (PCa) is the most common cancer in men in Western countries [1], and the number of males diagnosed with PCa in Asia is increasing dramatically [2]. In 2014, the Prostate Imaging-Reporting and Data System version 2 (PI-RADS v2) was published to simplify and standardize the terminology and content of radiology reports [3, 4]. The system classifies all imaging characters into 5 points to diagnose PCa, from 1 point, which has a very low probability, to 5 points, which has a very high probability [3]. However, the PI-RADS score is not perfect because its negative predictive value (NPV) is unstable [5]. Hence, coordinating imaging with other laboratory data is worth considering.

Prostate-specific antigen (PSA) screening is widely used to assess PCa, despite its false-positive rate and its overtreatment because of its inferior accuracy. The cut-off point of 4 ng/mL is not sufficient to evaluate the risk of PCa [6]. However, it is still the first index used to detect the presence of PCa [7]. PSA density (PSAD) and the free/total PSA ratio (f/t PSA), clinical statistics available from PSA, are considered more sensitive in predicting PCa [8, 9].

To date, few studies have combined PI-RADS v2 with clinical data to improve PCa detection [10, 11]. Washino S et al. combined PSAD as the only independent variable and used the detection rate as the sole evaluation index [10]. To estimate PCa more comprehensively, our study attempts to establish a new model with additional clinical variables by quantifying those results and thus providing the clinician with a convenient and simple model to diagnose PCa and even its biological malignancy.

Imitating PI-RADS v2, our study stratified PSA, PSAD and f/t PSA and gave each level a point. Then, a simple, leaner accumulating model was built, aiming at utilizing available data thoroughly, improving the diagnostic accuracy of PCa and ultimately decreasing the number of unnecessary biopsies.

## Methods

### Patient population

This retrospective study was performed at our institution. Institutional review board approval and informed consent were obtained. Related data of patients who had standard prostate magnetic resonance imaging (MRI) examinations for any reason between January 2014 and December 2017 were collected, including imaging results, the results of biochemical examinations, and pathological reports. The inclusion criteria were as follows: (i) all patients underwent standardized prostate multiparametric MRI (mpMRI) before drug, biopsy or surgical therapy, (ii) serum examination was performed before treatment, and (iii) transrectal ultrasonography and 12-core prostate biopsy with pathological results were performed in the next 2 weeks after mpMRI. Of the 1039 patients who had undergone prostate mpMRI, 357 patients were eligible for the study. Clinically significant PCa (CS PCa) was indicated by a maximum cancer core length ≥ 4 mm and/or a Gleason grade ≥ 3 + 4 [12].

### MRI

All patients underwent MRI examinations on 3.0 T GE equipment (DISCOVER MR750 GEHCGEHC) using a multichannel vitro coil. Turbo spin-echo T2-weighted imaging (repetition time msec/echo time msec, 4291–4569/95.9–101.2; section thickness, 3–4 mm; intersection gap, 0 mm; field of view, 200 × 200 mm; matrix, 352 × 352), diffusion-weighted imaging (DWI, repetition time msec/echo time msec, 4000/57–59; section thickness, 3–4 mm; intersection gap, 0 mm; field of view, 370 × 370 mm; matrix, 128 × 160), and apparent diffusion coefficient and dynamic contrast-enhanced imaging (repetition time msec/echo time msec, 4.3/1.9–2.0; section thickness, 3–4 mm; intersection gap, 0 mm; field of view, 320 × 320 mm; matrix, 320 × 224; dose 0.1 mmol/kg standard gadolinium-based contrast agent; injection rate: 2–3 cc/sec) were performed. The b value of DWI was 1500 s/mm^2^. The imaging was read by two independent radiologists who were trained through the PI-RADS v2 criterion and blinded to clinical data to decrease bias in reading the results. If the conclusions were not concordant, the final score was determined by a senior radiologist who specialized in abdominal radiology for more than 30 years. T2-weighted imaging and DWI were the dominant determining sequences for the central and peripheral zones, respectively. Prostate volume was measured in T1-weighted imaging, and a formula multiplying length by width by height by 0.52^3^ was applied.

### Pathology

The prostate biopsies were taken transrectally using an automatic biopsy gun and a 12 + X-G needle under ultrasound guidance (six in the peripheral zone, six in the transitional zone, X in the suspicious zone). TRUS-guided biopsy was combined with TRUS-guided targeted biopsy and cognitive MRI fusion-guided targeted biopsy. Prostate surgery included radical prostatectomy and transurethral resection of the prostate. The specimens that were obtained as described above were assessed by experienced pathologists.

### Statistical analyses

Clinical data included PSA, f/t PSA and PSAD. The classification of these variables referred to published papers that were based on large populations. PSA values were split into 4 levels: the cut-off points were 4, 10 and 20 ng/mL [13]. This study defined PSA < 4 ng/mL as level 1, 4–10 ng/mL as level 2, 10–20 ng/mL as level 3 and PSA greater than 20 ng/mL as level 4. Likewise, 0.1, 0.19 and 0.23 ng/mL/mL of PSAD were used to stratify patients into 4 planes [14, 15]. From the lower to upper layer, levels 1 to 4 were set for each plane. For f/t PSA, 0.14, 0.18, and 0.24 were defined as the cut-off scores [16]. An explicit figure was not given if PSA was greater than 1000 ng/mL, and we defined the f/t PSA as 0.001 in these situations. In contrast, level 1 was assigned for f/t PSA more than 0.24, and levels 2, 3, and 4 were listed in descending order by f/t PSA. Regarding the pathological results, the Gleason score (GS) was in accordance with the malignancy of the lesion [17]. According to the prognosis, GS was divided into four-tiered groupings: < 6 was defined as group 1, 6 as group 2, 7 as group 3, and 8 to 10 as group 4 [18–22].

Imaging data were combined with serum results to build accumulating models. Optimal weights of each level of quota were found to obtain the most satisfactory model. The following equations were derived based on the logistic regression equation and correlation coefficient. Thus, thirteen models were established to mutually compare to find a simple and satisfactory PCa accumulating system as follows:

- model 1: PI-RADS v2 + level of PSAD
- model 2: PI-RADS v2 + level of f/t PSA
- model 3: PI-RADS v2 + level of PSA
- model 4: PI-RADS v2 + level of PSAD + level of f/t PSA
- model 5: PI-RADS v2 + level of PSAD + level of PSA
- model 6: PI-RADS v2 + level of PSAD + level of f/t PSA + level of PSA
- model 7: 2 × PI-RADS v2 + 3 × level of PSAD
- model 8: 2 × PI-RADS v2 + 3 × level of PSAD + level of f/t PSA
- model 9: 2 × PI-RADS v2 + 3 × level of PSAD + level of PSA
- model 10: 2 × PI-RADS v2 + 3 × level of PSAD + level of f/t PSA+ level of PSA.
- model 11: 2 × PI-RADS v2 + 2 × level of PSAD + level of f/t PSA
- model 12: 2 × PI-RADS v2 + 2 × level of PSAD + level of PSA
- model 13: 2 × PI-RADS v2 + 2 × level of PSAD + level of f/t PSA + level of PSA.

The best-fit receiver operating characteristics of thirteen models were calculated. Univariate and multivariate logistic regression analyses were performed. The relationship between the scores of the accumulating model and GS was analysed by one-way ANOVA. All data were analysed in SPSS 2 3.0, and *p* < 0.05 indicated statistical significance.

## Results

Of the 357 patients involved in the study, 48.7% (174/357) had benign prostatic hyperplasia (group 1), 51.3% (183/357) were diagnosed with PCa, and 45.7% (163/357) had CS PCa. Among patients with PCa, 18.6% (34/183) had GS 3 + 3 PCa (group 2), 40.4% (74/183) had GS 7 (group 3), and 41.0% (75/183) had GS 8–10 (group 4). Of GS 3 + 3, 14 had a maximum cancer core length ≥ 4 mm. The patient characteristics are presented in Table 1. The medians and interquartile ranges of age, PSA, f/t PSA and PSAD were 68 (63–74), 11.06 (6.79–21.34), 0.13 (0.08–0.19) and 0.24 (0.15–0.56), respectively. In nonparametric correlations, the Kendall’s tau_b coefficients were as follows: between PSAD and f/t PSA, 0.422; between PSAD and PSA, 0.626; and between PSA and f/t PSA, 0.253. The variable levels of PSA, PSAD, f/t PSA and PI-RASD v2 were clearly related to the presence of PCa and CS PCa, especially PI-RADS v2 and the level of PSAD. The levels of PSA and f/t PSA were rejected in the PCa diagnosis in the logistic regression (Table 2). In a head-to-head-comparison between each category, the performances of levels of each variable are presented in Table 2.

**Table 1:** The patients’ characteristics

Variable	Vaule
	median
age, years	68
PSA ng/mL	11.06
f/t PSA	0.13
PSAD ng/mL/mL	0.24
Gleason score	frequency
BPH	174
score 6	34
score 7	74
score 8	47
score 9	22
score 10	6
PI-RADSv2 score	
1–2	154
3	35
4–5	168

*IQR* interquartile range, *mpMRI* multiparametric magnetic resonance imaging, *PI-RADS v2* The Prostate Imaging– Reporting and Data System Version 2, *BPH* benign prostatic hyperplasia

**Table 2:** Univariate and multivariate logistic regression analyses to detect PCa and CS PCa

	PCa								CS PCa							
	univariate analysis				multivariate analysis				univariate analysis				multivariate analysis			
	OR	95% Confidence Interval		P	OR	95% Confidence Interval		P	OR	95% Confidence Interval		P	OR	95% Confidence Interval		P
		Lower	Upper			Lower	Upper			Lower	Upper			Lower	Upper	
PI-RADS v2																
1–2	–	–	–	–	–	–	–	–	–	–	–	–	–	–	–	–
3	1.36	0.596	3.102	0.465	1.214	0.474	3.113	0.686	1.241	0.462	3.335	0.668	1.051	0.337	3.272	0.932
4	3.024	2.275	4.019	< 0.001	2.398	1.745	3.296	< 0.001	3.775	2.78	5.126	< 0.001	3.29	2.316	4.674	< 0.001
5	5.854	2.993	11.448	< 0.001	4.352	2.148	8.817	< 0.001	7.074	3.599	13.905	< 0.001	5.241	2.54	10.811	< 0.001
PSAD (ng/mL/mL)																
< 0.1	–	–	–	–	–	–	–	–	–	–	–	–	–	–	–	–
0.1–0.19	4.234	1.191	15.051	0.026	3.505	0.723	16.982	0.119	3.8	0.826	17.482	0.086	2.171	0.307	15.373	0.428
0.19–0.23	2.099	1.087	4.053	0.027	3.237	1.026	10.213	0.045	2.132	0.974	4.666	0.058	4.787	0.974	23.536	0.054
≥ 0.23	3.97	2.622	6.01	< 0.001	1.904	1.063	3.411	0.03	4.185	2.564	6.83	< 0.001	2.087	1.089	4	0.027
f/t PSA																
≥ 0.24	–	–	–	–	–	–	–	–	–	–	–	–	–	–	–	–
0.18–0.24	2.597	0.915	7.375	0.073	3.703	0.621	22.072	0.151	3.141	0.945	10.442	0.062	6.748	0.628	72.518	0.115
0.14–0.18	1.493	0.88	2.533	0.137	0.845	0.383	1.866	0.677	1.795	0.984	3.275	0.056	1.124	0.396	3.193	0.827
< 0.14	2.272	1.674	3.084	< 0.001	1.39	0.907	2.129	0.131	2.409	1.686	3.442	< 0.001	1.662	0.959	2.879	0.07
PSA (ng/ml)																
< 4	–	–	–	–	–	–	–	–	–	–	–	–	–	–	–	–
4–10	3.755	1.062	13.277	0.04	0.967	0.225	4.153	0.964	4.071	0.91	18.21	0.066	0.802	0.134	4.802	0.809
10–20	2.608	1.379	4.932	0.003	0.563	0.186	1.708	0.31	2.847	1.343	6.035	0.006	0.62	0.18	2.14	0.45
≥ 20	3.154	2.03	4.9	< 0.001	0.208	0.045	1.062	0.051	3.585	2.147	5.987	< 0.001	0.333	0.072	1.54	0.159

**Fig. 1:**
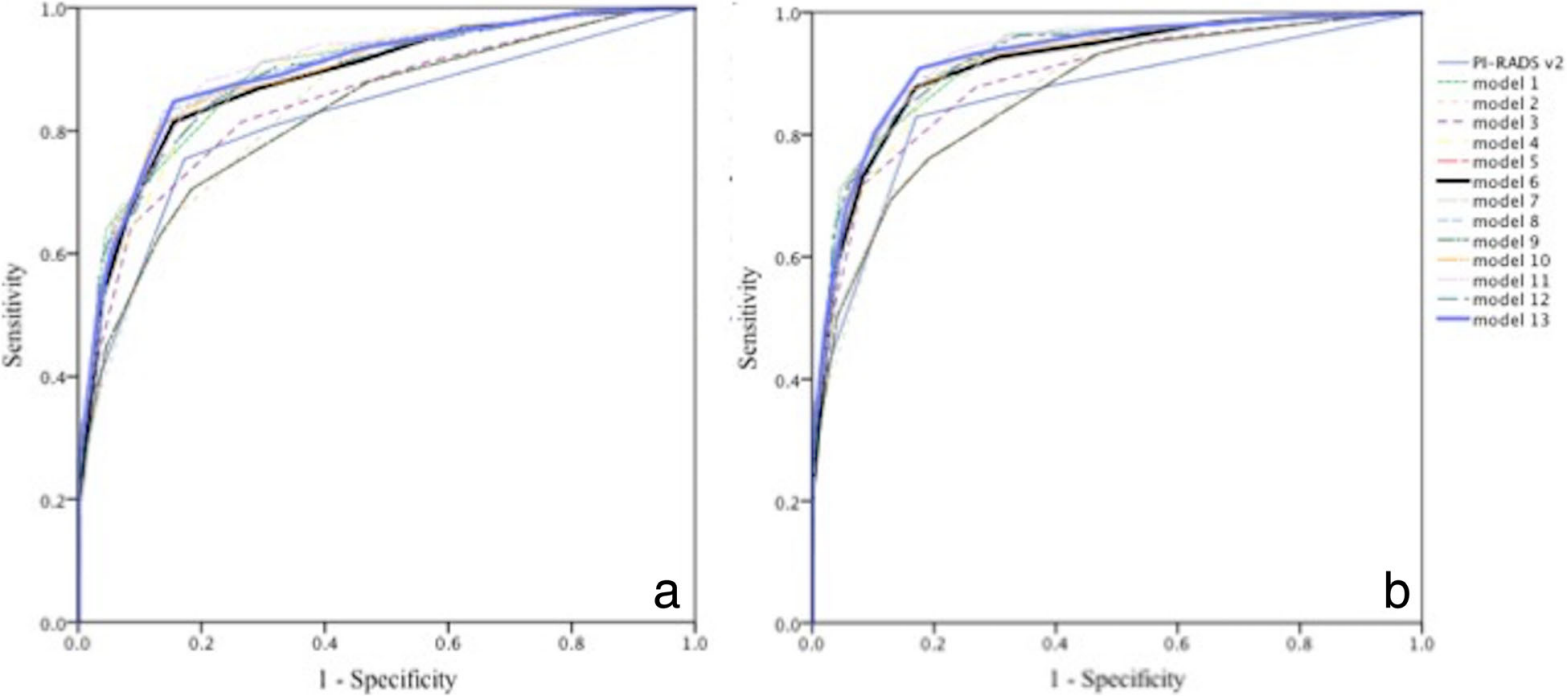
ROC curves of PI-RADS v2 and model 1–13. ROC curves of PI-RADS v2 and model 1–13 for predicting the presence of PCa (**a**) and CS PCa (**b**)

The receiver operating characteristic curves of models 1 to 13 were calculated (Fig. 1a, b). To diagnose PCa, it appeared that the area under the curve increased with additional clinical variables added, with PI-RADS v2 as the base. All models had more capacity to distinguish the absence or presence of CS PCa compared to PCa diagnosis. The characteristics of the 13 models are presented in Table 3.

**Table 3:** The characteristic of differernt ROC curves of model PI-RADS v2 and model 1 to 13 in diagnosing PCa and CS PCa

Test Result Variable(s)	Area		Asymptotic 95% Confidence Interval				sensitivity		specificity		PPV		NPV	
			Lower Bound		Upper Bound									
	Pca	CS PCa	PCa	CS PCa	PCa	CS PCa	Pca	CS PCa	PCa	CS PCa	PCa	CS PCa	PCa	CS PCa
PIRADS V2	0.821	0.86	0.777	0.82	0.866	0.901	0.758	0.834	0.828	0.83	0.822	0.805	0.766	0.856
model 1	0.891	0.922	0.858	0.894	0.925	0.95	0.716	0.791	0.891	0.892	0.873	0.86	0.749	0.836
model 2	0.849	0.874	0.81	0.839	0.887	0.909	0.705	0.767	0.793	0.794	0.782	0.758	0.719	0.802
model 3	0.847	0.889	0.807	0.854	0.887	0.923	0.814	0.877	0.736	0.732	0.764	0.733	0.79	0.877
model 4	0.891	0.916	0.858	0.887	0.924	0.945	0.918	0.914	0.649	0.778	0.734	0.776	0.883	0.915
model 5	0.877	0.91	0.841	0.88	0.912	0.94	0.847	0.773	0.753	0.881	0.783	0.846	0.824	0.822
model 6	0.884	0.913	0.849	0.882	0.918	0.943	0.814	0.877	0.845	0.83	0.847	0.813	0.812	0.89
model 7	0.892	0.922	0.859	0.894	0.925	0.95	0.836	0.896	0.799	0.784	0.814	0.777	0.822	0.899
model 8	0.896	0.924	0.863	0.896	0.929	0.951	0.842	0.89	0.822	0.84	0.832	0.824	0.831	0.901
model 9	0.886	0.918	0.852	0.888	0.92	0.947	0.842	0.877	0.78	0.804	0.798	0.79	0.823	0.886
model 10	0.892	0.921	0.858	0.893	0.925	0.95	0.836	0.865	0.821	0.856	0.832	0.834	0.847	0.896
model 11	0.898	0.926	0.866	0.899	0.931	0.953	0.88	0.933	0.799	0.773	0.821	0.776	0.863	0.932
model 12	0.889	0.922	0.855	0.894	0.923	0.95	0.847	0.828	0.782	0.866	0.803	0.839	0.829	0.857
model 13	0.889	0.925	0.862	0.898	0.928	0.953	0.847	0.908	0.845	0.825	0.852	0.813	0.84	0.813

As shown in Fig. 1a, models 6 and 13 were good. When scores of model 6 ≥ 11 were considered positive, the sensitivity, specificity, positive predictive value (PPV) and negative predictive value (NPV) for PCa were 0.814 (149/183), 0.845 (147/174), 0.847 (149/176) and 0.812 (147/181), respectively, compared to 0.754 (138/183), 0.828 (144/174), 0.821 (138/168) and 0.762 (144/189), respectively, for PI-RDAS v2. For CS PCa assessment, the cut-off point was 12, with sensitivity, specificity, PPV and NPV of 0.877 (143/163), 0.830 (161/194), 0.813 (143/176) and 0.890 (161/181), respectively, compared to 0.843 (136/163), 0.830 (161/194), 0.805 (136/169) and 0.856 (161/188), respectively, for PI-RDAS v2. In other words, among the 189 people with PI-RADS v2 scores of 1–3, 23.8% (45/189) PCa patients were missed, and 17.2% (30/168 PI-RADS v2 score 4–5) were false positives and underwent unnecessary biopsy. In model 6, 18.8% (34/181) were missed, and 15.3% (27/176) were false positives. In total, 11 patients were correctly diagnosed, and 3 were prevented from incorrect diagnosis when PI-RADS v2 was incorrect.

Model 13 showed a stronger capacity in diagnosing PCa. At a cut-off point of 18, the sensitivity, specificity, PPV and NPV were 0.847 (155/183), 0.845 (147/174), 0.852 (155/182) and 0.84 (147/175), respectively. To assess CS PCa, the cut-off point was 18, with sensitivity, specificity, PPV and NPV of 0.908 (148/163), 0.825 (160/194), 0.813 (148/182) and 0.914 (160/175), respectively.

**Fig. 2:**
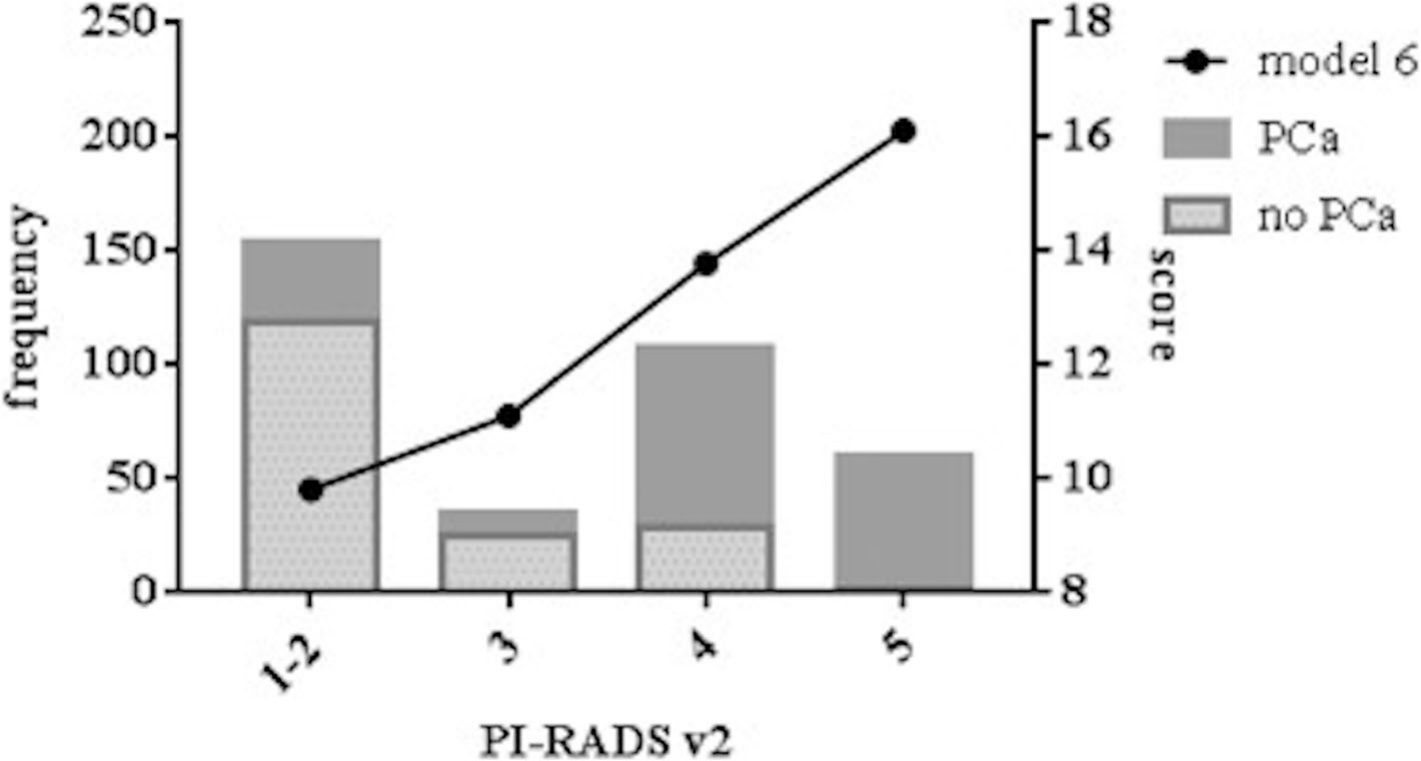
Relationship between model 6 and PI-RADS v2. Tendency of model 6 in pace with PI-RADS v2 and the distributions of PCa among PI-RADS v2 scores

The scores of model 6 and PI-RADS v2 are presented in Fig. 2. The score of model 6 increased as the GS increased. The mean scores of different four-tiered groupings exhibited significant differences (*p* < 0.001, Table 4). The mean values and 95% confidence intervals for the means of GS groups 1–4 were 10.20 (9.63–10.40), 12.03 (11.19–12.87), 14.12 (13.60–14.64) and 15.44 (15.09–15.79). The scores among GS 8–10 had no significant differences (*p* = 0.055).

**Table 4:** Scores of the model 6 in four-tiered Gleason score groupings

Gleason score	N	Mean	Std. Deviation	Std. Error	95% Confidence Interval for Mean		Minimum	Maximum
					Lower Bound	Upper Bound		
0 (group 1)	174	10.02	2.59	0.2	9.63	10.4	5	16
6 (group 2)	34	12.03	2.42	0.41	11.19	12.87	7	17
7 (group 3)	74	14.12	2.24	0.26	13.6	14.64	6	17
8–10 (group 4)	75	15.44	1.51	0.17	15.09	15.79	11	17
Total	357	12.2	3.26	0.17	11.86	12.54	5	17

## Discussion

Our study revealed that PI-RADS v2, PSA, f/t PSA and PSAD were significant predictors of PCa and CS PCa. In addition, our study established a new PCa accumulating model, which may assess the risk of PCa noninvasively. However, the scores for model 6 had no significant difference among GS 8 to 10.

In accordance with previous studies, PI-RADS v2 itself is efficient in diagnosing PCa. When the PI-RADS v2 score ≥ 4 was supposed to be positive, it had a sensitivity of 85–88% and a specificity of 55–71% for PCa [23]. However, because its specificity was low and its NPV was unstable [5], some people were misdiagnosed. In previous studies, a PI-RADS v2 score of 1–2 rarely yielded PCa, while the connection between PI-RADS v2 score 3 and the presence of PCa was uncertain [24, 25]. Our study found that the detection rates of PCa using PI-RADS v2 scores of 1–2, 3, and 4–5 were 22.7% (35/154), 28.6% (10/35) and 82.1% (138/168), respectively. In other words, 45 patients were missed when PI-RADS v2 score ≥ 4 was supposed to be positive, and 30 were over-diagnosed.

Serum PSA is the most common index to detect and decide the absence or presence of PCa and to monitor its aggressiveness [26]. In clinical practice, PSA is a reference to determine whether to perform a biopsy. However, the specificity of PSA in PCa detection is unsatisfactory. For people with PSA between 4 and 10 ng/mL, only a quarter suffered PCa [27]. As a result, many men without cancer underwent unnecessary biopsies, PCa cases were often detected, and CS PCa cases were sometimes missed [28, 29]. When PSA > 20 ng/ml, up to 84.2% (80/95) suffered from PCa in our study, and among these, 11.3% (9/80) were excluded from PCa in mpMRI. To improve the diagnostic accuracy, other serum indexes, such as f/t PSA and PSAD, are often used to assist PCa diagnosis. The f/t PSA combination increased the specificity of early detection compared to PSA alone [30]. Specificity could be improved threefold with stable sensitivity by incorporating f/t PSA into predictive factors [31]. The cut-off point of 0.18 has been widely used in clinical applications [16]. Few studies have explored the value of PSAD [32, 33]. In recent studies, patients with a PSAD of < 0.15 ng/mL/mL may avoid unnecessary biopsies [11]. Furthermore, PSAD had a positive influence on CS PCa diagnosis [33]. Previous studies reported the relationship between PI-RDAS v2 and PSA [11, 34]. According to their results, PSA and f/t PSA were not independent factors in logistic regression analysis. PSAD, as an independent index, was combined with PI-RADS v2 to explain its function in PCa detection. In our study, in patients with PI-RADS v2 scores ≥4 and/or PSAD ≥0.15/ng/ml/ml, the sensitivity, specificity, PPV and NPV were 61.4, 88.9, 95.6 and 36.8%, respectively. It showed high PPV when it adhered to these criteria, with low NPV. Collectively, it is worth trying to coordinate PI-RADS with PSA, f/t PSA and PSAD for predicting PCa.

Our study emphasizes the importance of comprehensively assessing both the imaging and clinical data of patients. Imitating PI-RADS v2, this study adopts an accumulating model and, to our knowledge, is the first study to use an accumulating system in diagnosing PCa. The study stratified all variables into several levels and put weight on each level with different scores to simplify and quantify the assessment of PCa. When the method of quantifying and accumulating the data is used, the subject and individual experience effects decrease. Building an accumulating system that is suitable and that can be applied in clinical settings may be meaningful. For convenient clinical application, model 6 is recommended, and model 13 is recommended to diagnose PCa more accurately. Model 6 shows satisfactory diagnostic capacity and is convenient, which not only enhances the accuracy of the diagnosis of PCa and CS PCa but also quantifies the process of assessing PCa. Although PSA and f/t PSA were rejected in logistic regression, they are helpful in increasing the specificity and sensitivity in the diagnosis of PCa and CS PC (Fig. 1). Thus, PSA and f/t PSA were included in the predictive model whether they were independent predictors or not. We also found that when the effects of PI-RADS v2 and PSAD were emphasized, the efficiencies of PSA and f/t PSA decreased (models 8 to 10), which may result from the level of PSAD has a strong relation to the level of PSA (K = 0.626) and f/t PSA (K = 0.422).

The head-to-head-comparison between each category showed the odds radios of each level. In univariate logistic regression analyses, higher levels of each variable had higher approximate risks of PCa when *p* < 0.05 indicated statistical significance. The 95% confidence intervals of level 2 of PSAD, f/t PSA and PSA were too broad to evaluate the risk, possibly because it was an unclear zone for detecting PCa and CS PCa and needed more specific classifications.

Further, the scores of model 6 are related to GS. Previous studies have reported that PI-RADS v2 had no significant correction with GS distribution [35], and to our knowledge, there is no research reporting the relationship between GS and the combination of PI-RADS v2 and PSA. In our study, the scores for model 6 increased in the four-tiered groupings. The scores for GS 8 to 10 had no significant differences. This may be because there were not enough cases of these GS to analyse, and the stratification criterion of the study does not consider dividing data that are far away from the range of normal values. In this study, it did not classify GS 7 into 3 + 4 and 4 + 3 because having few cases would lead to result shifting. However, the study is still valuable, as the prognoses of GS at 8–10 can be classified into the same group [17]. This result may be helpful in assessing aggressiveness in non-invasive testing.

In all, our study describes a new way to predict the presence of PCa using an intuitive and objective score to balance the difference in efficiency among all parameters. When the case is negative according to PI-RADS v2, our study gives a simple reference regarding whether to perform a biopsy on the basis of the level of PSA, f/t PSA and PSAD in clinical work.

However, the study has some limitations. First, our study is retrospective, and patient selection bias exists. There was no case of a score of 1 in PI-RADS v2 because the patients’ mean age in this study was higher than 60. The signal we observed on T2WI was not homogeneous in these elderly patients. We paid no attention to the role of extreme values of clinical data. At our institution, the test would not provide an explicit figure if the PSA level was greater than 1000 ng/mL, and we defined the f/t PSA as 0.001 in these situations. However, this would not upgrade the f/t PSA group. Further studies may include more clinical data, such as age, race and family history. It would be better to stratify PSA along with age to accumulate scores [36, 37].

## Conclusions

Our study takes advantage of PSA, f/t PSA and PSAD by combining these variables with PI-RADS v2 to establish a new accumulating model that increases the accuracy of the primary diagnosis of PCa and may be helpful in the clinical decision to perform a biopsy.

## Abbreviations

CS PCa: Clinically significant prostate cancer
DWI: Diffusion weighted imaging
f/t PSA: Free/total PSA ratio
GS: Gleason score
MR: Magnetic resonance
PCa: Prostate cancer
PI-RADS v2: Prostate Imaging-Reporting and Data System Version 2
PSA: Prostate specific antigen
PSAD: PSA density
